# The Plasma B-Type Natriuretic Peptide Levels Are Low in Males with Stable Ischemic Heart Disease (IHD) Compared to Those Observed in Patients with Non-IHD: A Retrospective Study

**DOI:** 10.1371/journal.pone.0108983

**Published:** 2014-10-31

**Authors:** Kosuke Minai, Takayuki Ogawa, Makoto Kawai, Kimiaki Komukai, Toshikazu Tanaka, Kazuo Ogawa, Tomohisa Nagoshi, Satoshi Arase, Satoshi Morimoto, Yasunori Inoue, Hiroshi Sekiyama, Akihiro Urabe, Seiichiro Matsuo, Kenichi Hongo, Michihiro Yoshimura

**Affiliations:** Division of Cardiology, Department of Internal Medicine, The Jikei University School of Medicine, Tokyo, Japan; Mayo Clinic, United States of America

## Abstract

**Objective:**

Although the plasma B-type natriuretic peptide (BNP) level is a marker of heart failure, it is unclear whether BNP *per se* plays a pivotal role for pathogenic mechanisms underlying the development of ischemic heart disease (IHD). In this study, we retrospectively examined the plasma BNP levels in stable patients with IHD and compared to stable patients with cardiovascular diseases other than IHD.

**Methods:**

The study population was 2088 patients (1698 males and 390 females) who were admitted to our hospital due to IHD (n = 1,661) and non-IHD (n = 427) and underwent cardiac catheterization. Measurements of the hemodynamic parameters and blood sampling were performed.

**Results:**

The plasma BNP levels were significantly lower in the IHD group than in the non-IHD group (p<0.001). The multiple regression analysis examining the logBNP values showed that age, a male gender, low left ventricular ejection fraction, low body mass index, serum creatinine, atrial fibrillation and IHD *per se* were significant explanatory variables. When the total study population was divided according to gender, the plasma BNP levels were found to be significantly lower in the IHD group than in the non-IHD group among males (p<0.001), but not females (p = NS). Furthermore, a multiple logistic regression analysis of IHD showed the logBNP value to be a significant explanatory variable in males (regression coefficient: −0.669, p<0.001), but not females (p = NS).

**Conclusions:**

The plasma BNP levels were relatively low in stable patients with IHD compared with those observed in stable patients with non-IHD; this tendency was evident in males. Perhaps, the low reactivity of BNP is causally associated with IHD in males. We hope that this study will serve as a test of future prospective studies.

## Introduction

A-type natriuretic peptide and B-type natriuretic peptide (BNP), also known as atrial and brain natriuretic peptides, respectively, are cardiac hormones with a wide range of potent biological effects, including vasodilation, natriuresis and inhibition of the renin-angiotensin-aldosterone and sympathetic nervous systems [1]–[8]. BNP is selectively secreted from the ventricles, and the magnitude of secretion also varies as a function of the severity of heart failure. The BNP value is thus used as a biochemical marker of heart failure.

In addition, we previously reported that, in patients with acute myocardial infarction (AMI), the time course of the changes in the plasma BNP levels just after the onset of AMI exhibited a biphasic pattern, with the first peak occurring approximately 24 hours after the onset and the second peak 3–5 days after the onset. The second peak of plasma BNP was more marked in severe cases of AMI with heart failure [9].

The plasma immunoreactive BNP level is a sensitive biochemical marker of heart failure; although the precise molecular forms of BNP and its precursors have been discussed from different angles [10], [11]. BNP increases the cyclic guanosine monophosphate (cGMP) levels [1]. cGMP activated by BNP is protective against cellular injury as it increase particulate guanylate cyclases [12]. BNP may also be beneficial for suppressing the progression of heart failure and atherosclerosis [13], [14]. If the plasma BNP level is insufficiently increased for any reason, heart failure and atherosclerosis are likely to advance; therefore, low plasma BNP level, even in patients with high risk factors, may play a causative role in the pathogenetic mechanisms underlying the development of IHD and heart failure. However, the discussion regarding this issue has been insufficient to date, although genetic variations of BNP and related molecules have previously been reported [15]–[21]. It is also unclear whether the plasma BNP level actually varies according to the individual and whether individual changes in this parameter are associated with disease. Nevertheless, there were a few early reports showing dysregulation of ANP and BNP in patients prone to developing hypertension and other cardiovascular disease [22]–[24].

Here, we hypothesized that BNP contributes to a pathogenetic mechanism of IHD, and retrospectively examined a possible contribution by comparing the BNP levels between stable patients with IHD and stable patients with cardiovascular diseases other than IHD.

## Methods

### Study design

The study population was 2088 consecutive patients admitted to the Jikei University Hospital from January 2008 through January 2012 in whom cardiac catheterization, including left ventriculography, and blood sampling for the plasma BNP levels were performed and reviewed. The baseline patient characteristics, including the clinical parameters and the biochemical data, were collected retrospectively from the medical records. The study protocol was approved by the ethics committee of the Jikei University School of Medicine (24–355; 7121).

### Recruitment of stable IHD patients and non-IHD patients in this study

The stable IHD patients consisted of 1,661 patients who had coronary stenosis newly diagnosed by angiography, or who had a medical history of coronary artery disease, such as previous myocardial infarction, post-percutaneous coronary intervention and coronary artery bypass graft. A total of 762 patients underwent only one cardiac catheterization, while 359 patients underwent catheterization twice, 50 patients three times, five patients four times, one patient five times and one patient underwent catheterization six times. In order to avoid a selection bias by choosing among the plural data in one patient, we used all consecutive data in this analysis (n = 1661). Patients with coronary spastic angina were included in the stable IHD group if the disease activity of coronary spasm was not clinically high. Coronary spasm was diagnosed by the acetylcholine provocation test. Patients with previously diagnosed coronary spastic angina were also included in the stable IHD group. In contrast, patients requiring urgent catheter intervention for AMI were excluded from this study because the plasma BNP levels are rapidly and extensively changes during the acute phase of AMI [9]. Similarly, patients with acute coronary syndrome were excluded from the stable IHD group in this study. In addition, Also, patients with chest pain syndrome of unknown origin and those with suspected microvascular angina were excluded from the stable IHD group because we were unable to appropriately define cardiac ischemia in such patients. Detailed information of the patients in the stable IHD group were summarized in Table 1.

**Table 1:** Baseline characteristics and the past medical history and medication regimen of the study subjects.

	Total			Female			Male		
	Non-IHD (n = 427)	IHD (n = 1,661)	P Value	Non-IHD (n = 144)	IHD (n = 246)	P-Value	Non-IHD (n = 283)	IHD (n = 1,415)	P-Value
**Age (yrs ± SD)**	63.1±13.1	65.2±10.5	P<0.01	67.5±13.5	70.4±10.2	P<0.05	60.9±12.3	64.4±10.3	P<0.001
**Male (%)**	66.3	85.1	P<0.001						
**Body mass index (kg/m2 ± SD)**	23.2±3.6	24.3±3.4	P<0.001	21.9±3.2	23.0±3.5	P<0.01	23.7±3.6	24.5±3.4	P<0.001
**Current smoker (%)**	21.8	23.2	NS	8.3	12.6	NS	28.6	25.1	NS
**Current+past smoker (%)**	53.4	69.8	P<0.001	19.4	28.5	P<0.05	70.7	77.0	P<0.05
**Hypertension (%)**	64.6	76.5	P<0.001	66.0	77.6	P<0.05	64.0	76.3	P<0.001
**Diabetes Mellitus (%)**	28.8	45.1	P<0.001	27.1	42.7	P<0.01	29.7	45.5	P<0.001
**Dyslipidemia (%)**	45.7	75.9	P<0.001	43.1	74.0	P<0.001	47.0	76.3	P<0.001
**s-Cr (mg/dl ± SD)**	1.6±2.4	1.6±2.3	P = 0.001	1.6±2.5	1.5±2.3	NS	1.6±2.3	1.6±2.3	NS
**HbA1c (% ± SD)**	5.8±1.1	6.0±1.1	P<0.001	5.7±1.1	5.9±0.8	NS	5.8±1.1	6.1±1.1	P<0.001
**BNP (pg/ml ± SD)**	310.2±650.0	157.6±401.1	P<0.001	319.5±650.4	303.2±668.2	NS	305.5±650.9	132.3±327.4	P<0.001
**HD & CAPD (%)**	10.8	9.3	NS	13.2	13.0	NS	9.5	8.7	NS
**LVEF (% ± SD)**	56.0±14.4	57.7±10.8	P<0.05	59.3±12.3	59.5±11.6	NS	54.4±15.1	57.4±10.7	P = 0.001
**Heart rate (beat/min ± SD) (At LVG)**	74.0±16.4	70.6±13.5	P<0.001	73.3±14.0	72.5±14.3	NS	74.4±17.6	70.3±13.4	P<0.001
**LVEDP (mmHg ± SD) (At PreLVG)**	16.5±7.2	15.5±6.3	P<0.05	16.4±7.0	16.1±6.9	NS	16.6±7.3	15.5±6.1	P<0.05
**LVESVI (ml/m^2^ ± SD)**	36.5±25.1	28.9±16.3	P<0.001	31.7±19.8	27.3±17.0	P<0.05	39.0±27.2	29.1±16.2	P<0.001
**Prior MI (%)**	0	38.5	P<0.001	0	35.8	P<0.001	0	38.9	P<0.001
**Prior PCI (%)**	0	44.6	P<0.001	0	35.8	P<0.001	0	46.1	P<0.001
**Prior CABG (%)**	0	12.5	P<0.001	0	15.9	P<0.001	0	12.0	P<0.001
**Coronary spastic angina**	0	8.5	P<0.001	0	12.6	P<0.001	0	7.8	P<0.001
**Prior valve repair (%)**	4.9	0.7	P<0.001	7.6	1.6	P<0.01	3.5	0.5	P<0.001
**Valvular heart disease (%)**	30.2	3.4	P<0.001	38.2	6.9	P<0.001	26.1	2.8	P<0.001
**Congenital heart disease (%)**	5.2	0.36	P<0.001	8.3	0.4	P<0.001	3.5	0.4	P<0.001
**Cardiomyopathy (%)**	22.7	2.0	P<0.001	20.8	3.3	P<0.001	23.7	1.8	P<0.001
**AF (%)**	20.6	5.5	P<0.001	19.4	4.9	P<0.001	21.2	5.7	P<0.001
**Calcium-channel blockers (%)**	39.1	60.6	P<0.001	43.1	59.8	P = 0.001	37.1	60.8	P<0.001
**ACE-inhibitors (%)**	15.0	21.3	P<0.01	14.6	18.3	NS	15.2	21.8	P<0.05
**Angiotensin Receptor Blockers (%)**	37.9	41.0	NS	44.4	42.7	NS	34.6	40.7	NS (P = 0.056)
**Nitrates (%)**	9.1	26.6	P<0.001	8.3	28.5	P<0.001	9.5	26.2	P<0.001
**Nicorandil (%)**	4.2	20.4	P<0.001	4.2	23.2	P<0.001	4.2	19.9	P<0.001
**Beta-blockers (%)**	18.7	34.4	P<0.001	20.8	28.9	NS (P = 0.081)	17.7	35.3	P<0.001
**Statins (%)**	23.0	59.3	P<0.001	29.9	57.7	P<0.001	19.4	59.6	P<0.001
**Fibrates (%)**	3.3	3.4	NS	1.4	1.2	NS	4.2	3.8	NS
**Diuretics (%)**	32.1	19.1	P<0.001	38.2	28.5	P<0.05	29.0	17.5	P<0.001
**Oral Hypoglycemic Agents (%)**	11.5	23.5	P<0.001	9.7	18.3	P<0.05	12.4	24.5	P<0.001
**insulin (%)**	5.2	9.9	P<0.01	6.9	10.6	NS	4.2	9.8	P<0.01
**Number of vessels disease (0–3)**	0	0.95±0.97		0	0.96±0.96		0	0.95±097	

s-Cr, Serum creatinine; BNP, B-type natriuretic peptide; HD & CAPD, Hemodialysis & continuous ambulatory peritoneal dialysis; LVEF, Left ventricular ejection fraction; LVEDP, Left ventricular end-diastolic pressure; LVESVI, Left ventricular end-systolic volume Index; MI, Myocardial Infarction; PCI, Percutaneous coronary intervention; CABG, Coronary artery bypass graft; AF, Atrial Fibrillation.

The non-IHD group consisted of 427 patients; all of those had organic heart diseases such as valvular heart diseases, cardiomyopaty, congenital heart disease and others. All non-IHD patients were clinically stable and admitted to the hospital for evaluation of their underlying cardiac disease. A total of 406 patients underwent cardiac catheterization one time, while nine patients were catheterized twice and one patient was catheterized three times. We used all consecutive data in this analysis just as we did in the IHD group (n = 427). We excluded patients suffering from acute heart failure at the time of cardiac cathetherization and blood sampling. Precise information including underlying cardiac disorders was also summarized in Table 1.

### Measurement of the plasma BNP levels and other parameters

Whole blood (5 ml) was collected in tubes containing potassium EDTA (1 mg/ml blood). The plasma BNP level was determined by an enzyme-linked immunosorbent assay (non-extracted) using an antibody against human BNP (Shionogi Co. Ltd., Tokyo, Japan). Blood sampling was performed immediately before and after the cardiac catheterization whenever possible. The average interval between catheterization and blood sampling was 5.21±8.94 days; 1384 (66.3%) of patients had blood collected for BNP within 72 hours post catheterization. Of these patients, 830 underwent blood collection at the time of the procedure (during, immediately before or just after cardiac catheterization), 1185 underwent blood collection within 24 hours, and 1342 underwent blood collection within 48 hours.

The BMI was calculated as the body weight (kg) divided by the square of the height (m). Hypertension, diabetes mellitus and dyslipidemia were defined as described previously [25].

### Statistical analysis

Continuous variables were expressed as the means ± SD. Categorical variables were expressed as percentages. Comparisons between groups were performed using Pearson's chi-square test for categorical variables and the Mann-Whitney U test or Student's t-test for continuous variables, where appropriate. To achieve a normal distribution, the BNP value was log-transformed before the analysis. To assess the dependent determinants of the log BNP, a multiple regression analysis was performed after the simple regression analysis. Age, gender (0 for females and 1 for males), IHD (0 for non-IHD and 1 for IHD), atrial fibrillation (0 for non-AF and 1 for AF), the LVEF, age, BMI, and s-Cr were included as variables in the multiple regression analysis. In addition, a gender-segregated multiple logistic regression analysis was performed to determine predictive factors for IHD using age, LVEF, BMI, s-Cr, atrial fibrillation (0 for non-AF and 1 for AF), and logBNP. A value of p<0.05 was considered to be statistically significant for all data that were statistically analyzed using the SPSS software package, version 21.0 (SPSS Inc., Chicago, IL).

## Results

### Clinical characteristics of the study subjects

Table 1 shows the clinical characteristic and the past medical history and medication regimen of the total 2088 patients.

High age, male gender, high BMI, smoking, hypertension, diabetes mellitus and dyslipidemia were more frequently seen in the IHD group than in the non-IHD group. The s-Cr was significantly higher in the non-IHD group than in the IHD group. The heart rate, LVESVI and LVEDP were lower in the IHD group than in the non-IHD group. The LVEF was slightly higher in the IHD group than in the non-IHD group. In this study, AF was more frequently detected in the non-IHD group than in the IHD group, which was due to the higher prevalence of valvular heart disease and cardiomyopathy in the non-IHD groups (precise data not shown in the Results).

The clinical characteristics are shown by gender in Table 2. It is important to compare the clinical characteristics between the females and males in the IHD group. For example, the LVEF was lower and the s-Cr was higher in males, whereas the age was lower and BMI values were higher in this group. With regard to the medical treatments, diuretics were used more frequently in females, and beta-blockers were used more frequently in males, while angiotensin-converting enzyme inhibitors/angiotensin II receptor blockers (ARBs) were used equally in females and males. Every patient had been prescribed the classes and doses of drugs based on his or her individual condition; therefore, it is difficult to compare the drugs more precisely. Nevertheless, the frequency in use and the amount used of major drugs such as enalapril, cavedilol or spironolactones was equivalent between females and males with or without IHD, while only the frequency of use and amount used of furosemide was significantly higher in males than in females (precise data not shown).

**Table 2:** Comparisons between female and male with and without IHD.

	Non-IHD			IHD		
	Female (n = 144)	Male (n = 283)	P-Value	Female (n = 246)	Male (n = 1,415)	P-Value
**Age (yrs ± SD)**	67.5±13.5	60.9±12.3	P<0.001	70.4±10.2	64.4±10.3	P<0.001
**Body mass index (kg/m2 ± SD)**	21.9±3.2	23.7±3.6	P<0.001	23.0±3.5	24.5±3.4	P<0.001
**Current smoker (%)**	8.3	28.6	P<0.001	12.6	25.1	P<0.001
**Current+past smoker (%)**	19.4	70.7	P<0.001	28.5	77.0	P<0.001
**Hypertension (%)**	66.0	64.0	NS	77.6	76.3	NS
**Diabetes Mellitus (%)**	27.1	29.7	NS	42.7	45.5	NS
**Dyslipidemia (%)**	43.1	47.0	NS	74.0	76.3	NS
**s-Cr (mg/dl ± SD)**	1.6±2.5	1.6±2.3	P<0.001	1.5±2.3	1.6±2.3	P<0.001
**HbA1c (% ± SD)**	5.7±1.1	5.8±1.1	NS	5.9±0.8	6.1±1.1	P<0.05
**BNP (pg/ml ± SD)**	319.5±650.4	305.5±650.9	P<0.05	303.2±668.2	132.3±327.4	P<0.001
**HD & CAPD (%)**	13.2	9.5	NS	13.0	8.7	P<0.05
**LVEF (% ± SD)**	59.3±12.3	54.4±15.1	P<0.001	59.5±11.6	57.4±10.7	P<0.01
**Heart rate (beat/min ± SD) (At LVG)**	73.3±14.0	74.4±17.6	NS	72.5±14.3	70.3±13.4	P<0.05
**LVEDP (mmHg ± SD) (At Pre LVG)**	16.4±7.0	16.6±7.3	NS	16.1±6.9	15.5±6.1	NS
**LVESVI (ml/m^2^ ± SD)**	31.7±19.8	39.0±27.2	P<0.01	27.3±17.0	29.1±16.2	NS
**Prior myocardial infarction (%)**	0	0		35.8	38.9	NS
**Prior PCI (%)**	0	0		35.8	46.1	P<0.01
**Prior CABG**	0	0		15.9	12.0	NS
**Coronary spastic angina**	0	0		12.6	7.8	P<0.05
**Prior valve repair (%)**	7.6	3.5	NS	1.6	0.5	P<0.05
**Valvular heart disease (%)**	38.2	26.1	P = 0.01	6.9	2.8	P = 0.001
**Congenital heart disease (%)**	8.3	3.5	P<0.05	0.4	0.4	NS
**Cardiomyopathy (%)**	20.8	23.7	NS	3.3	1.8	NS
**AF (%)**	19.4	21.2	NS	4.9	5.7	NS
**Calcium-channel blockers (%)**	43.1	37.1	NS	59.8	60.8	NS
**ACE-inhibitors (%)**	14.6	15.2	NS	18.3	21.8	NS
**Angiotensin Receptor Blockers (%)**	44.4	34.6	P<0.05	42.7	40.7	NS
**Nitrates (%)**	8.3	9.5	NS	28.5	26.2	NS
**Nicorandil (%)**	4.2	4.2	NS	23.2	19.9	NS
**Beta-blockers (%)**	20.8	17.7	NS	28.9	35.3	P<0.05
**Statins (%)**	29.9	19.4	P<0.05	57.7	59.6	NS
**Fibrates (%)**	1.4	4.2	NS	1.2	3.8	P<0.05
**Diuretics (%)**	38.2	29.0	NS	28.5	17.5	P<0.001
**Oral Hypoglycemic Agents (%)**	9.7	12.4	NS	18.3	24.5	P<0.05
**insulin (%)**	6.9	4.2	NS	10.6	9.8	NS
**Number of vessels disease (0–3)**	0	0		0.96±0.96	0.95±097	NS

s-Cr, Serum creatinine; BNP, B-type natriuretic peptide; HD & CAPD, Hemodialysis & continuous ambulatory peritoneal dialysis; LVEF, Left ventricular ejection fraction; LVEDP, Left ventricular end-diastolic pressure; LVESVI, Left ventricular end-systolic volume Index; PCI, Percutaneous coronary intervention; CABG, Coronary artery bypass graft;AF, Atrial Fibrillation.

### Comparison of the plasma BNP levels between the non-IHD and IHD groups and statistical analysis of the logBNP values in the total study population

**Figure 1:**
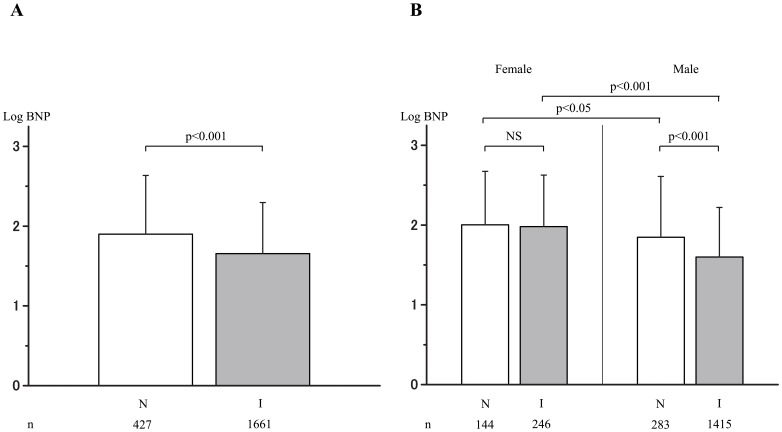
Comparison of the logBNP levels between the non-IHD and IHD groups. A: In the overall population. B: In males and females. N: non-ischemic heart disease group (non-IHD); I: ischemic heart disease group (IHD). BNP: B-type natriuretic peptide.

The plasma BNP levels were significantly lower in the IHD group than in the non-IHD group as shown in Figure 1-A. In order to identify factors contributing to the difference in the plasma BNP levels between the IHD and non-IHD groups among the total study population, statistical analyses was performed, as shown in Table 3 and Table 4. Table 3 is the results of the simple regression analysis of the logBNP values using clinical factors as explanatory variables. Table 4 shows the results of the subsequent multiple regression analysis. Consequently, age, gender, BMI, s-Cr, LVEF, AF, and IHD were found to be significant determinants of the logBNP value. Among these factors, it is noteworthy that IHD *per se* was significantly and inversely correlated with the logBNP value.

**Table 3:** The results of the simple regression analyses for the log BNP in all patients (n = 2088).

Explanatory variable	Regression coefficient	Standard regression coefficient	P	95%CI
**Age**	0.016	0.270	<0.001	0.014∼0.019
**Gender**	−0.348	−0.203	<0.001	−0.420∼−0.276
**BMI**	−0.047	−0.247	<0.001	−0.055∼−0.039
**s-Cr**	0.119	0.415	<0.001	0.108∼0.130
**LVEF**	−0.027	−0.478	<0.001	−0.030∼−0.025
**AF**	0.597	0.251	<0.001	0.499∼0.696
**IHD**	−0.243	−0.147	<0.001	−0.314∼−0.173

BMI, Body mass index; s-Cr, Serum creatinine; LVEF, Left ventricular ejection fraction; AF, Atrial fibrillation; IHD, Ischemic heart disease.

**Table 4:** The results of the multiple regression analyses for the log BNP in all patients (n = 2088).

Explanatory variable	Regression coefficient	Standard regression coefficient	P	95%CI	VIF
**Age**	0.015	0.249	<0.001	0.013∼0.017	1.125
**Gender**	−0.272	−0.159	<0.001	−0.327∼−0.217	1.120
**BMI**	−0.015	−0.081	<0.001	−0.022∼−0.009	1.128
**s-Cr**	0.095	0.333	<0.001	0.086∼0.104	1.061
**LVEF**	−0.024	−0.414	<0.001	−0.026∼−0.022	1.097
**AF**	0.325	0.137	<0.001	0.249∼0.401	1.107
**IHD**	−0.115	−0.069	<0.001	−0.168∼−0.061	1.131

BMI, Body mass index; s-Cr, Serum creatinine; LVEF, Left ventricular ejection fraction; AF, Atrial fibrillation; IHD, Ischemic heart disease.

### Comparison of the plasma BNP levels between the non-IHD and IHD groups based on gender

Figure 1B shows that females with and without IHD had higher levels of plasma BNP compared to males with and without IHD. The plasma BNP levels were similar in females with and without IHD (p = NS, actually p = 0.725). In contrast, the plasma BNP level was lower in males with IHD compared to males without IHD (p<0.001).

### The results of the multiple logistic regression analysis for the determination of IHD

**Figure 2:**
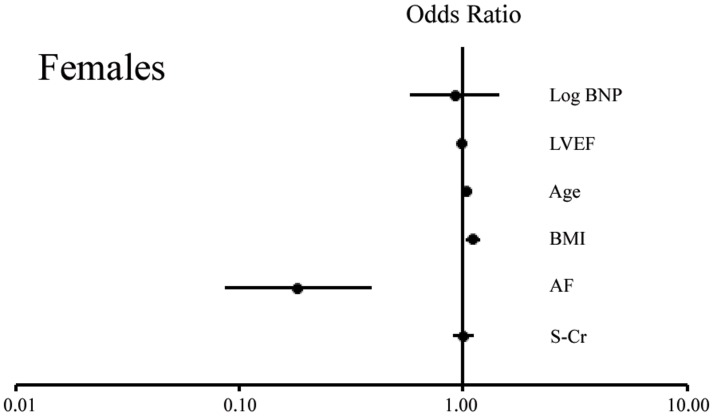
The Forest Plot displaying the odds ratio about risk of IHD in a females. BNP, B-type natriuretic peptide; LVEF, Left ventricular ejection fraction; BMI, Body mass index; AF, Atrial fibrillation; IHD, Ischemic heart disease; s-Cr, Serum creatinine.

**Figure 3:**
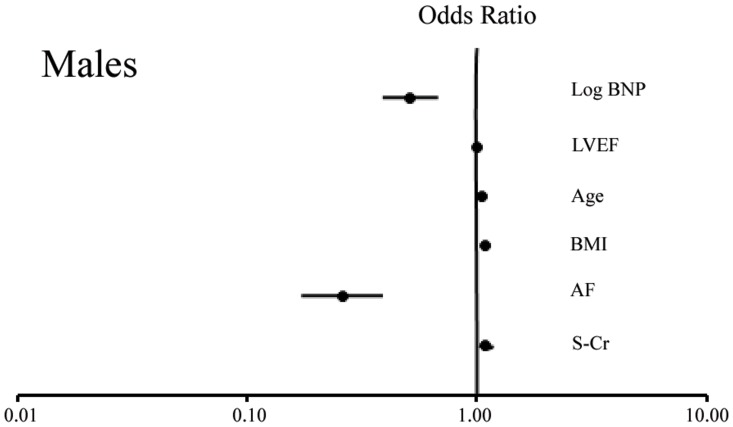
The Forest Plot displaying the odds ratio about risk of IHD in a males. BNP, B-type natriuretic peptide; LVEF, Left ventricular ejection fraction; BMI, Body mass index; AF, Atrial fibrillation; IHD, Ischemic heart disease; s-Cr, Serum creatinine.

We finally examined the factors determining the presence of IHD; a multiple logistic regression analysis was performed for the determination of IHD by using the logBNP, LVEF, age, BMI, AF and s-Cr as explanatory variables in females and males. As shown in Tables 5 and Figure 2 (Females), and Table 6, and Figure 3 (Males), the trend between females and males was similar for age (significant in each gender), BMI (significant in each gender), AF (significant in each gender) and LVEF (not significant in each gender); however, the s-Cr was not significant in females (p = NS) but was significant in males (p<0.05). This result may suggest that chronic kidney disease is a more important risk factor for IHD in males than in females. It is noteworthy that the logBNP was not significantly associated with IHD in females (p = NS, actually p = 0.719), but was significantly and inversely associated with IHD in males (Regression coefficient: −0.669, p <0.001). Thus, the logBNP may only correlate with IHD in males, and may have a possible causative role, although it is difficult to distinguish between cause and effect based on the present analysis.

**Table 5:** The results of the logistic regression analysis for IHD in females.

Explanatory variable	Regression coefficient	P	Odds ratio	Odds 95% CI
**Log BNP**	−0.085	NS (p = 0.719)	0.919	0.579∼1.457
**LVEF**	−0.014	NS (p = 0.232)	0.986	0.963∼1.009
**Age**	0.027	<0.01	1.028	1.007∼1.048
**BMI**	0.101	<0.01	1.106	1.034∼1.183
**AF**	−1.703	<0.001	0.182	0.086∼0.388
**s-Cr**	−0.002	NS (p = 0.965)	0.998	0.901∼1.105

BNP, B-type natriuretic peptide; LVEF, Left ventricular ejection fraction; BMI, Body mass index; AF, Atrial fibrillation; IHD, Ischemic heart disease; s-Cr, Serum creatinine.

**Table 6:** The results of the logistic regression analysis for IHD in males.

Explanatory variable	Regression coefficient	P	Odds ratio	Odds 95% CI
**Log BNP**	−0.669	<0.001	0.512	0.388∼0.677
**LVEF**	−0.007	NS (p = 0.318)	0.993	0.981∼1.006
**Age**	0.049	<0.001	1.050	1.037∼1.064
**BMI**	0.080	<0.001	1.083	1.039∼1.128
**AF**	−1.351	<0.001	0.259	0.172∼0.389
**s-Cr**	0.087	0.01	1.091	1.021∼1.167

BNP, B-type natriuretic peptide; LVEF, Left ventricular ejection fraction; BMI, Body mass index; AF, Atrial fibrillation; IHD, Ischemic heart disease; s-Cr, Serum creatinine.

### Sub-analysis based on the presence or absence of AF

AF was found to be significantly associated with non-IHD in both females and males, as shown in Table 5 and Figure 2 (Females) and Table 6 and Figure 3 (Males). Therefore, an additional analysis would be necessary, and the study population was subsequently divided based on the presence or absence of AF: the AF (+) group and the AF (−) group. There were no significant differences in the plasma BNP levels between the non-IHD and IHD groups among the AF (+) or AF (−) females; however, the plasma BNP levels were significantly lower in the IHD group than in the non-IHD group among AF(+) or AF(−) males. The subgroup analysis of AF therefore suggested that the tendency toward a low plasma BNP level in IHD males was not affected by AF.

## Discussion

In the beginning of the study, we found the plasma BNP levels to be specifically lower in the stable patients with IHD than in the stable patients with non-IHD among the total study population. In addition, the multiple regression analysis clearly showed for the first time that IHD *per se* is an independent determinant of the plasma BNP level, in addition to other known factors, such as age, male gender, low BMI, s-Cr, low LVEF and AF [26]–[31]. Furthermore, when the total study population was divided by gender, significant differences were identified between females and males. For example, it is noteworthy that the plasma BNP levels were significantly lower in the IHD group than in the non-IHD group among males only. In this analysis, the gender-difference observed in the plasma BNP levels in the IHD group was prominent even in consideration of the diverse clinical backgrounds. Therefore, unknown factors likely contributed to the observed gender difference. Next, by a multiple logistic regression analysis conversely performed using IHD as an objective variable and the logBNP as one of the explanatory variables, IHD was inversely determined by the logBNP only in males, but not in females. Hence the statistical analysis clarified that there is a substantial association between IHD *per se* and low plasma BNP level in males only. It is difficult to distinguish between cause and effect based on the present analyses; however, low plasma levels of BNP may be causally associated with IHD in males. In other words, the factor ‘low reactivity of the plasma BNP level’ may be a new risk factor for IHD in males. This way of thinking is supported by the previous reports showing that a deficiency of ANP and BNP was causatively associated with hypertension and other cardiovascular diseases [22]–[24].

BNP is biologically active and increases the cGMP levels. BNP is thus beneficial for suppressing the progression of heart failure, and probably atherosclerosis [13], [14]. If the BNP level is insufficiently increased, low plasma BNP levels may have a causative role in IHD and heart failure. The reasons for the comparatively low plasma BNP levels or deteriorated response of BNP in IHD are unknown at present. Various factors such as neurohormones or genetic factors associated with gender differences may be linked to the current results. In addition, it is unknown whether a reduction of the synthesis or an augmented clearance of BNP affected the current results. As a next step, we need to examine the precise mechanisms responsible for the low plasma BNP levels seen only in male patients with IHD. The findings of the present and future studies may be able to provide a new strategy to prevent IHD and heart failure by increasing the endogenous natriuretic peptides using agents such as neutral endopeptidase inhibitors.

It is interesting to note a gender difference in the contribution of the plasma BNP level to the pathogenesis of IHD, as a gender difference have generally been reported to be negligible in patients with heart failure to date [27]. The results of this study may therefore help to answer the remaining questions regarding the impact of gender differences in the setting of IHD [32]–[37]. At present, it remains unknown whether IHD is the only or one of several cardiovascular diseases associated with the gender difference in the plasma BNP levels.

In general, the measurement of the plasma BNP levels is useful for early monitoring of asymptomatic ventricular dysfunction in patients with high risk factors. A value <18.4 pg/ml is considered to be ideal, and less than 40 pg/ml is considered to be within the normal range for patients without cardiac organic disorders visiting the hospital [27]. However, in this study, a considerable number of patients with a low EF (<55%) and low plasma BNP levels (<40 pg/ml) was seen in the IHD group compared with the non-IHD group among males; 153 patients (10.81%) vs. 16 patients (5.65%), p<0.01. On the other hand, there were few female patients with the same conditions; only two patients (0.81%) in the IHD group and nine patients (6.25%) in the non-IHD group, p<0.01 (precise data not shown in the Results). This indicates that clinicians should take care not to underestimate the degree of heart failure even if the plasma BNP levels are relatively low (less than 40 pg/ml) in male patients with IHD.

It is known that the BNP level increases with inflammation [38]. In this study, we examined the serum C-reactive protein (CRP) levels between 396 patients with IHD and 136 patients with non-IHD via blood samples obtained on the day of the catheterization. The average CRP level was 0.670±1.653 (95%CI: 0.390–0.950) in the non-IHD group and 0.521±1.109 (95%CI: 0.412–0.631) in the IHD group, which was not significantly different (p = 0.239) (precise data not shown in the Results). Therefore, we thought that the inflammation status would not have influenced the present results.

To avoid a selection bias, the study population was consecutively and non-selectively recruited; therefore, all plural data from one patient were used in this analysis. We essentially performed the analysis by using 1,661 IHD patients and 427 non-IHD patients. However, by way of caution, we performed a similar statistical analysis by using only one dataset from each patient (1178 IHD patients and 416 non-IHD patients) who underwent cardiac catheterization for the first time during this protocol. As a result, the plasma BNP levels were found to be significantly lower in the IHD group than in the non-IHD group (p<0.001), the same as in the full dataset. In addition, when the total study population was divided by gender, the plasma BNP levels were significantly lower in the IHD group than in the non-IHD group only in males (p<0.001), but not in females (p = NS). Thus, the findings of the full dataset and the data from just individual cases were the same.

Finally, this is only a pilot retrospective study of the possible contribution of a low plasma BNP level to the pathogenesis of IHD. Therefore, a prospective study is needed to examine whether male patients with very low levels of plasma BNP, even under conditions of high risk factors, are more likely to develop IHD, for example, by comparing the rates of acute ischemic attack, the progression of IHD and the severity of stenosis in IHD patients grouped by the plasma BNP level.

## Study Limitation

First, the number of females was small compared with that of males (1698 males and 390 females); thus, it is difficult to draw any definitive conclusions regarding the gender difference in the contribution of the plasma BNP level to IHD. A larger study including more females is required to arrive at an absolute conclusion. Second, while the plasma BNP level was the primary outcome measurement of the study, the samples were collected on a wide range intervals, and up to 33.7% of the subjects underwent blood collection more than 72 hours after cardiac catheterization. It is ideal to perform simultaneous sampling with hemodynamic measurements during cardiac catheterization; however, this was a retrospective study, and blood sampling for the purpose of measuring the plasma BNP levels was performed in the clinical setting based on the discretion of the attending physician. For this reason, there was a time delay in blood sampling in some patients. However, no statistical differences were noted in the timing of sampling between females and males; the patients who underwent blood sampling after more than 72 hours included 125 females (32.1% of the total 390 females) and 579 males (34.1% of the total 1,698 males) (P = NS). Therefore, we do not believe that the time lag in sampling in some cases largely affected the present findings.

## Conclusions

The plasma BNP levels were relatively low in stable patients with IHD compared with that observed in the stable patients with non-IHD; this tendency was more evident in males. Perhaps, the low reactivity of BNP is causally associated with IHD in males. We hope this study serves as a test of a prospective study in the future.

## References

[1] Sudoh T, Kangawa K, Minamino N, Matsuo H (1988) A new natriuretic peptide in porcine brain. Nature 332: 78–81. 2964562 10.1038/332078a0

[2] Mukoyama M, Nakao K, Hosoda K, Suga S, Saito Y, et al (1991) Brain natriuretic peptide as a novel cardiac hormone in humans. Evidence for an exquisite dual natriuretic peptide system, atrial natriuretic peptide and brain natriuretic peptide. J Clin Invest 87: 1402–1412. 1849149 10.1172/JCI115146 PMC295184

[3] Yasue H, Yoshimura M, Sumida H, Kikuta K, Kugiyama K, et al (1994) Localization and mechanism of secretion of B-type natriuretic peptide in comparison with those of A-type natriuretic peptide in normal subjects and patients with heart failure. Circulation 90: 195–203. 8025996 10.1161/01.cir.90.1.195

[4] Omland T, Aakvaag A, Bonarjee VV, Caidahl K, Lie RT, et al (1996) Plasma brain natriuretic peptide as an indicator of left ventricular systolic function and long-term survival after acute myocardial infarction. Comparison with plasma atrial natriuretic peptide and N-terminal proatrial natriuretic peptide. Circulation 93: 1963–1969. 8640969 10.1161/01.cir.93.11.1963

[5] Tsutamoto T, Wada A, Maeda K, Hisanaga T, Maeda Y, et al (1997) Attenuation of compensation of endogenous cardiac natriuretic peptide system in chronic heart failure: prognostic role of plasma brain natriuretic peptide concentration in patients with chronic symptomatic left ventricular dysfunction. Circulation 96: 509–516. 9244219 10.1161/01.cir.96.2.509

[6] Richards AM, Nicholls MG, Espiner EA, Lainchbury JG, Troughton RW, et al (2003) B-type natriuretic peptides and ejection fraction for prognosis after myocardial infarction. Circulation 107: 2786–2792. 12771003 10.1161/01.CIR.0000070953.76250.B9

[7] Suzuki S, Yoshimura M, Nakayama M, Mizuno Y, Harada E, et al (2004) Plasma level of B-type natriuretic peptide as a prognostic marker after acute myocardial infarction: a long-term follow-up analysis. Circulation 110: 1387–1391. 15353502 10.1161/01.CIR.0000141295.60857.30

[8] Wang TJ, Larson MG, Levy D, Benjamin EJ, Leip EP, et al (2004) Plasma natriuretic peptide levels and the risk of cardiovascular events and death. N Engl J Med 350: 655–663. 14960742 10.1056/NEJMoa031994

[9] Morita E, Yasue H, Yoshimura M, Ogawa H, Jougasaki M, et al (1993) Increased plasma levels of brain natriuretic peptide in patients with acute myocardial infarction. Circulation 88: 82–91. 8319360 10.1161/01.cir.88.1.82

[10] Hawkridge AM, Heublein DM, Bergen HR III, Cataliotti A, Burnett JC Jr, et al (2005) Quantitative mass spectral evidence for the absence of circulating brain natriuretic peptide (BNP-32) in severe human heart failure. Proc Natl Acad Sci U S A 102: 17442–17447. 16293687 10.1073/pnas.0508782102 PMC1297688

[11] Nishikimi T, Okamoto H, Nakamura M, Ogawa N, Horii K, et al (2013) Direct immunochemiluminescent assay for proBNP and total BNP in human plasma proBNP and total BNP levels in normal and heart failure. PLoS One 8: e53233. 23365636 10.1371/journal.pone.0053233 PMC3554706

[12] Gorbe A, Giricz Z, Szunyog A, Csont T, Burley DS, et al (2010) Role of cGMP-PKG signaling in the protection of neonatal rat cardiac myocytes subjected to simulated ischemia/reoxygenation. Basic Res Cardiol 105: 643–650. 20349314 10.1007/s00395-010-0097-0

[13] Ogawa Y, Itoh H, Tamura N, Suga S, Yoshimasa T, et al (1994) Molecular cloning of the complementary DNA and gene that encode mouse brain natriuretic peptide and generation of transgenic mice that overexpress the brain natriuretic peptide gene. J Clin Invest 93: 1911–1921. 8182124 10.1172/JCI117182 PMC294298

[14] Nishikimi T, Maeda N, Matsuoka H (2006) The role of natriuretic peptides in cardioprotection. Cardiovasc Res 69: 318–328. 16289003 10.1016/j.cardiores.2005.10.001

[15] Costello-Boerrigter LC, Boerrigter G, Ameenuddin S, Mahoney DW, Slusser JP, et al (2011) The effect of the brain-type natriuretic peptide single-nucleotide polymorphism rs198389 on test characteristics of common assays. Mayo Clin Proc 86: 210–218. 21364112 10.4065/mcp.2010.0708 PMC3046941

[16] Szabo G, Molvarec A, Stenczer B, Rigo J Jr, Nagy B (2011) Natriuretic peptide precursor B gene (TTTC)(n) microsatellite polymorphism in pre-eclampsia. Clin Chim Acta 412: 1371–1375. 21514286 10.1016/j.cca.2011.04.012

[17] Ellis KL, Newton-Cheh C, Wang TJ, Frampton CM, Doughty RN, et al (2011) Association of genetic variation in the natriuretic peptide system with cardiovascular outcomes. J Mol Cell Cardiol 50: 695–701. 21276798 10.1016/j.yjmcc.2011.01.010

[18] Choquet H, Cavalcanti-Proenca C, Lecoeur C, Dina C, Cauchi S, et al (2009) The T-381C SNP in BNP gene may be modestly associated with type 2 diabetes: an updated meta-analysis in 49 279 subjects. Hum Mol Genet 18: 2495–2501. 19377085 10.1093/hmg/ddp169

[19] Poreba R, Poczatek K, Gac P, Poreba M, Gonerska M, et al (2009) SNP rs198389 (T-381 C) polymorphism in the B-type natriuretic peptide gene promoter in patients with atherosclerotic renovascular hypertension. Pol Arch Med Wewn 119: 219–224. 19413180

[20] Weber M, Burian M, Dragutinovic I, Moellmann H, Nef H, et al (2008) Genetic polymorphism of the type A human natriuretic peptide receptor (NPR-A) gene contributes to the interindividual variability in the BNP system. Eur J Heart Fail 10: 482–489. 18436476 10.1016/j.ejheart.2008.03.009

[21] Vassalle C, Andreassi MG, Prontera C, Fontana M, Zyw L, et al (2007) Influence of ScaI and natriuretic peptide (NP) clearance receptor polymorphisms of the NP System on NP concentration in chronic heart failure. Clin Chem 53: 1886–1890. 17890443 10.1373/clinchem.2007.088302

[22] Ferrari P, Weidmann P, Ferrier C, Dietler R, Hollmann R, et al (1990) Dysregulation of atrial natriuretic factor in hypertension-prone man. J Clin Endocrinol Metab 71: 944–951. 2144858 10.1210/jcem-71-4-944

[23] Belluardo P, Cataliotti A, Bonaiuto L, Giuffre E, Maugeri E, et al (2006) Lack of activation of molecular forms of the BNP system in human grade 1 hypertension and relationship to cardiac hypertrophy. Am J Physiol Heart Circ Physiol 291: H1529–1535. 16648193 10.1152/ajpheart.00107.2006

[24] Macheret F, Boerrigter G, McKie P, Costello-Boerrigter L, Lahr B, et al (2011) Pro-B-type natriuretic peptide(1-108) circulates in the general community: plasma determinants and detection of left ventricular dysfunction. J Am Coll Cardiol 57: 1386–1395. 21414536 10.1016/j.jacc.2011.01.005 PMC3927966

[25] Komukai K, Ogawa T, Yagi H, Date T, Sakamoto H, et al (2008) Decreased renal function as an independent predictor of re-hospitalization for congestive heart failure. Circ J 72: 1152–1157. 18577827 10.1253/circj.72.1152

[26] Nakane T, Kawai M, Komukai K, Kayama Y, Matsuo S, et al (2012) Contribution of extracardiac factors to the inconsistency between plasma B-type natriuretic peptide levels and the severity of pulmonary congestion on chest X-rays in the diagnosis of heart failure. Intern Med 51: 239–248. 22293797 10.2169/internalmedicine.51.6206

[27] Kawai M, Yoshimura M, Harada M, Mizuno Y, Hiramitsu S, et al (2013) Determination of the B-type natriuretic peptide level as a criterion for abnormalities in japanese individuals in routine clinical practice: the J-ABS Multi-Center Study (Japan Abnormal BNP Standard). Intern Med 52: 171–177. 23318845 10.2169/internalmedicine.52.8704

[28] Cataliotti A, Malatino LS, Jougasaki M, Zoccali C, Castellino P, et al (2001) Circulating natriuretic peptide concentrations in patients with end-stage renal disease: role of brain natriuretic peptide as a biomarker for ventricular remodeling. Mayo Clin Proc 76: 1111–1119. 11702899 10.4065/76.11.1111

[29] Redfield MM, Rodeheffer RJ, Jacobsen SJ, Mahoney DW, Bailey KR, et al (2002) Plasma brain natriuretic peptide concentration: impact of age and gender. J Am Coll Cardiol 40: 976–982. 12225726 10.1016/s0735-1097(02)02059-4

[30] Wang TJ, Larson MG, Levy D, Leip EP, Benjamin EJ, et al (2002) Impact of age and sex on plasma natriuretic peptide levels in healthy adults. Am J Cardiol 90: 254–258. 12127613 10.1016/s0002-9149(02)02464-5

[31] Knudsen CW, Riis JS, Finsen AV, Eikvar L, Muller C, et al (2004) Diagnostic value of a rapid test for B-type natriuretic peptide in patients presenting with acute dyspnoe: effect of age and gender. Eur J Heart Fail 6: 55–62. 15012919 10.1016/j.ejheart.2003.10.006

[32] Njolstad I, Arnesen E, Lund-Larsen PG (1996) Smoking, serum lipids, blood pressure, and sex differences in myocardial infarction. A 12-year follow-up of the Finnmark Study. Circulation 93: 450–456. 8565161 10.1161/01.cir.93.3.450

[33] Jousilahti P, Vartiainen E, Tuomilehto J, Puska P (1999) Sex, age, cardiovascular risk factors, and coronary heart disease: a prospective follow-up study of 14 786 middle-aged men and women in Finland. Circulation 99: 1165–1172. 10069784 10.1161/01.cir.99.9.1165

[34] Yarnell JW, Beswick AD, Sweetnam PM, Riad-Fahmy D (1993) Endogenous sex hormones and ischemic heart disease in men. The Caerphilly prospective study. Arterioscler Thromb 13: 517–520. 8466887 10.1161/01.atv.13.4.517

[35] Malkin CJ, Pugh PJ, Jones TH, Channer KS (2003) Testosterone for secondary prevention in men with ischaemic heart disease? Qjm 96: 521–529. 12881595 10.1093/qjmed/hcg086

[36] Schuit SC, Oei HH, Witteman JC, Geurts van Kessel CH, van Meurs JB, et al (2004) Estrogen receptor alpha gene polymorphisms and risk of myocardial infarction. Jama 291: 2969–2977. 15213208 10.1001/jama.291.24.2969

[37] Rod NH, Kristensen TS, Diderichsen F, Prescott E, Jensen GB, et al (2010) Cortisol, estrogens and risk of ischaemic heart disease, cancer and all-cause mortality in postmenopausal women: a prospective cohort study. Int J Epidemiol 39: 530–538. 20022927 10.1093/ije/dyp354

[38] Inoue T, Kawai M, Nakane T, Nojiri A, Minai K, et al (2010) Influence of low-grade inflammation on plasma B-type natriuretic peptide levels. Intern Med 49: 2659–2668. 21173540 10.2169/internalmedicine.49.4211

